# The Wire Twisting/Locking Technique to Facilitate Precise PDA Stent Delivery in Neonates with Ductal-Dependent Pulmonary Blood Flow

**DOI:** 10.1007/s00246-025-03852-2

**Published:** 2025-05-05

**Authors:** Marjan Hesari, Mercy Ng’eno, Brent M. Gordon, Laith AlShawabkeh, Justin R. Ryan, Clinton Fulk, Howaida El-Said

**Affiliations:** 1 Department of Pediatrics, University of California San Diego, La Jolla, CA USA; 2 Rady Children’S Hospital Division of Cardiology, San Diego, San Diego, CA USA; 3 Department of Cardiology, University of California San Diego, La Jolla, CA USA; 4 Helen and Will Webster Foundation 3 d Innovations Lab, Rady Children’S Hospital, San Diego, CA USA; 5 Department of Neurological Surgery, UC San Diego Health, La Jolla, CA USA

**Keywords:** PDA stenting, Twisting/locking technique, Neonatal intervention, Congenital heart disease

## Abstract

Patent ductus arteriosus (PDA) stenting is an established alternative to systemic-to-pulmonary artery shunts for neonates with ductal-dependent pulmonary blood flow, offering a less invasive approach. However, the procedure remains technically challenging and prone to complications. We evaluated the wire twisting/locking technique to improve precision and safety during PDA stenting. This retrospective study, approved by the UC San Diego Institutional Review Board, analyzed the application of the wire twisting/locking technique for PDA stenting between Jan 2021 and Dec 2024. Data were extracted from electronic medical records, including patient demographics, diagnoses, procedural details, and outcomes. The wire twisting/locking technique was applied in 18 PDA stenting procedures performed on 11 patients with complex cyanotic congenital heart disease. The procedures included initial PDA stenting (*n* = 10) and PDA stent re-interventions (*n* = 8), comprising four balloon dilations and four stent placements. The median age of initial stent replacement was 9 days (IQR: 8–90), and the median weight was 3.88 kg (IQR: 3.17–6.05). Access sites included carotid (*n* = 10), femoral (*n* = 4), axillary (*n* = 2), and umbilical arteries (n = 2). The median stent diameter was 2.05 mm (IQR: 1.5–2.4), and the median stent length was 24.35 mm (IQR: 22–30 mm). The median procedure time was 91 min (IQR: 62–130) and a median radiation dose of 154.73 Gycm^2^. All procedures were successful, with no vascular or pulmonary complications. Median discharge oxygen saturation was 85% (IQR: 82–89). The wire twisting/locking technique represents a promising modification for PDA stenting. Utilizing a dynamic soft wire and opting for a single long stent simplifies stent placement and minimizes procedural complications.

**Supplementary Information:**

The online version contains supplementary material available at 10.1007/s00246-025-03852-2.

## Background

Neonates with complex cyanotic congenital heart disease (CCHD) rely on the patent ductus arteriosus (PDA) for pulmonary perfusion after birth [1]. PDA stenting has emerged as a less invasive alternative to systemic pulmonary shunts, offering comparable or improved outcomes while reducing the need for cardiopulmonary bypass and enabling faster recovery [2–5]. However, PDA stenting remains technically challenging, with potential complications such as thrombosis, dissection, hemorrhage, and stent malposition [6–9]. Reinterventions are often necessary due to issues like intimal hyperplasia, stent fractures, or the need to prolong palliation until definitive repair [9–11]. The wire twisting/locking technique is previously described for outflow tract (RVOT) stenting [12]. More recently, Kato et al. adapted this technique for PDA stenting via femoral access, demonstrating its potential advantages in securing the guidewire and improving procedural success [13]. Here, we extend this approach using multiple access sites (carotid, axillary, umbilical, femoral), employing a dynamic BMW wire to optimize stent delivery in neonates. This study evaluates the technique’s efficacy and safety, addressing the need for precision in challenging PDA anatomies.

## Method

This is a retrospective case series analyzing clinical data from neonates with CCHD and duct-dependent pulmonary circulation who underwent PDA stenting using the wire twisting/locking technique at Rady Children's Hospital between January 2021 and December 2024. Institutional review board approval was obtained, and informed consent was waived for this study. All procedures followed institutional guidelines and adhered to the ethical standards.

### Inclusion Criteria

- Neonates undergoing wire twisting/locking for initial PDA stenting or re-intervention.

### Exclusion Criteria

- Use of the wire twisting/locking technique for non-PDA indications.
- PDA stenting procedures that did not involve the wire twisting/locking technique.

All cases were reviewed and approved in multidisciplinary team meetings before intervention. Patient demographics, clinical characteristics, procedural details, and follow-up data were retrieved from electronic medical records and securely stored in REDCap.

### PDA Morphology Classification

PDA morphology was classified based on the Congenital Cardiovascular Interventional Study Consortium (CCISC) classification, which categorizes PDAs according to origin and tortuosity (types 1–3). The PDA origin was further classified as arising from either the transverse aortic arch or the innominate artery. This classification system has been previously described and was applied in this study [14].

### Study Procedure

All procedures were performed in the catheterization laboratory under general anesthesia and biplane fluoroscopic guidance. Heparin was given to maintain an ACT of > 220, and intravenous antibiotics were administered.

### Access Site Selection

**Fig. 1.**
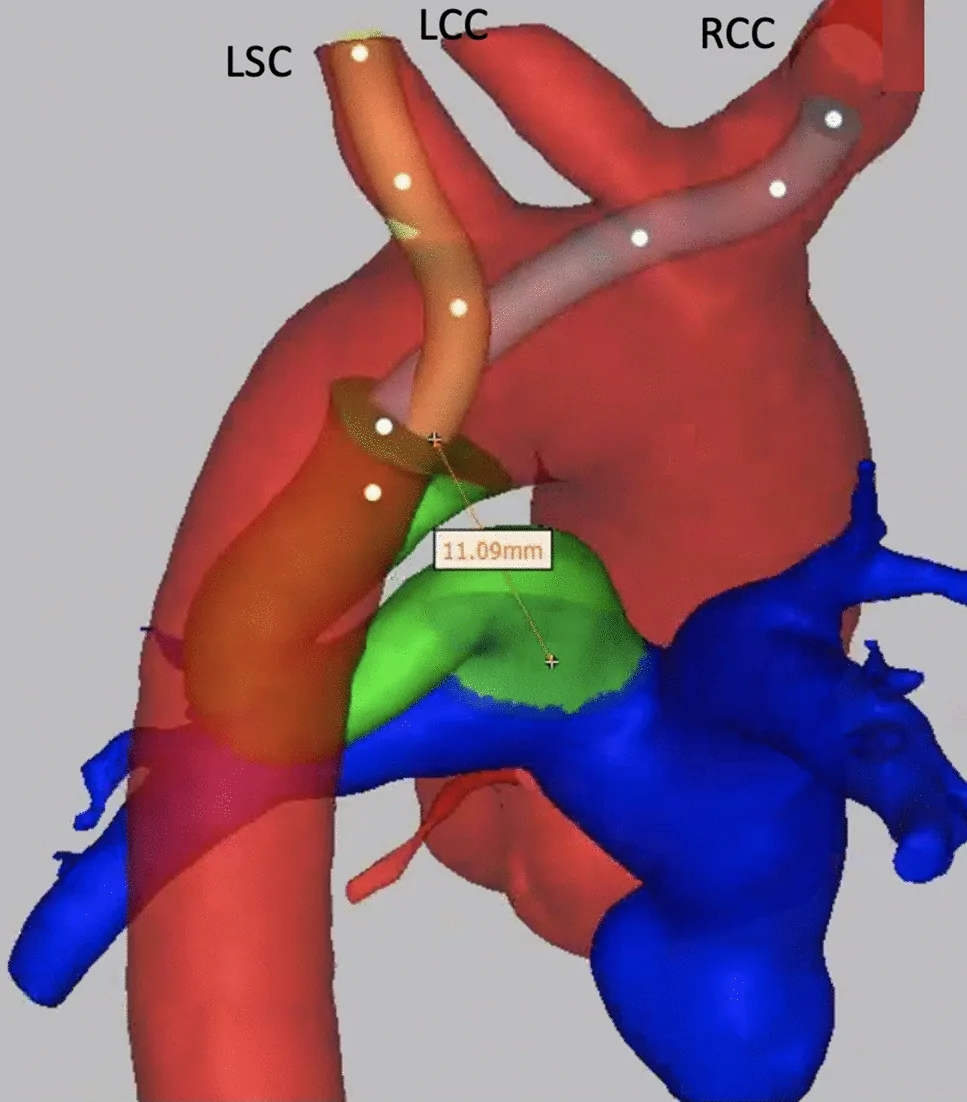
3D reconstruction from CT scan showing a posterior view of the aorta (red), pulmonary arteries (blue), and the PDA (green). The orange cylinder represents the trajectory from the left axillary artery to the PDA orifice, while the gray cylinder represents the trajectory from the right axillary artery. Notably, although the left axillary/left subclavian artery is anatomically closer to the PDA, the trajectory from the right common carotid artery aligns more directly with the PDA. The thin orange line illustrates the length of the PDA from the aortic to the pulmonary end if fully straightened, highlighting that the actual PDA length is much longer when accounting for its natural tortuosity. *LSC* left subclavian artery, *LCC* left common carotid artery, *RCC* right common carotid artery

All de novo PDA stent patients underwent a pre-procedural CT scan, which was used to determine both the appropriate stent length and optimal vascular access site. The choice of access site was guided by the trajectory of the PDA to the head and neck vessels. To better illustrate this process, we utilized a computer-generated cylindrical spline to simulate stent positioning. (Fig. 1).

Vascular access was obtained using the Seldinger technique rather than surgical cutdown for femoral, carotid, and axillary as has been previously described [15, 16]. The use of umbilical arterial access for PDA stenting has been adopted by our group recently. We utilize this approach when the trajectory of the PDA is favorable for a femoral route, allowing us to preserve the femoral artery and minimize vascular complication in pts where umbilical arterial access is still available.

### Prostaglandin Management Protocol Based on PDA Anatomy

Prostaglandin (PGE) management is tailored to the patient's PDA anatomy.

- Type I PDAs: PGE is discontinued prior to the procedure to allow the PDA to reach the desired size. The time required to achieve this size is recorded, and PGE is then restarted at 0.01 mcg/kg/min. Before the procedure, PGE is stopped again for the same number of hours it initially took to reach the desired PDA size.
- Type II and III PDAs: The PGE dose is reduced to 0.01 mcg/kg/min, and we typically avoid stopping it altogether. Our goal is to have an oxygen saturation between 70–85% prior to catheterization.

In our experience, tortuous PDAs (Type II and III) often have more tissue, which provide a better anchoring site for the stent. Thus, in many cases, we can maintain low-dose PGE without compromising stent positioning or stability. However, because every baby is different, these adjustments are made case by case, balancing the need for ductal patency and optimizing the procedural conditions.

## Procedure Details

### Wire and Catheter Insertion

A 4 Fr × 7 cm Prelude ideal sheath (Merit Medical, Salt Lake City, UT, USA) was inserted through the chosen access site (carotid, axillary, or femoral). Heparin was administered during the procedure to maintain ACT ~ 250. For umbilical access, a 4 F 45 cm flexor sheath (Cook Medical, Bloomington, IN, USA) with a handmade curve was utilized to navigate the tortuous pathway through the umbilical and iliac arteries. A 4 F-angled glide catheter (Terumo, Somerset, NJ, USA), 2.7 Fr renegade microcatheter (Boston Scientific, Marlborough, MA, USA), and 0.014"× 300 cm BMW wire (Abbott, Santa Clara, CA, USA) were placed through the sheath. The BMW wire and Renegade catheter were carefully navigated across the PDA or PDA stent in case of re-intervention and advanced into the distal pulmonary artery branch, either the left or right, mostly in the lower lobe, depending on the patient's anatomy. Care was taken to avoid advancing the sheath or the angled glide catheter into the PDA to minimize the risk of spasm or dissection.

**Fig. 2:**
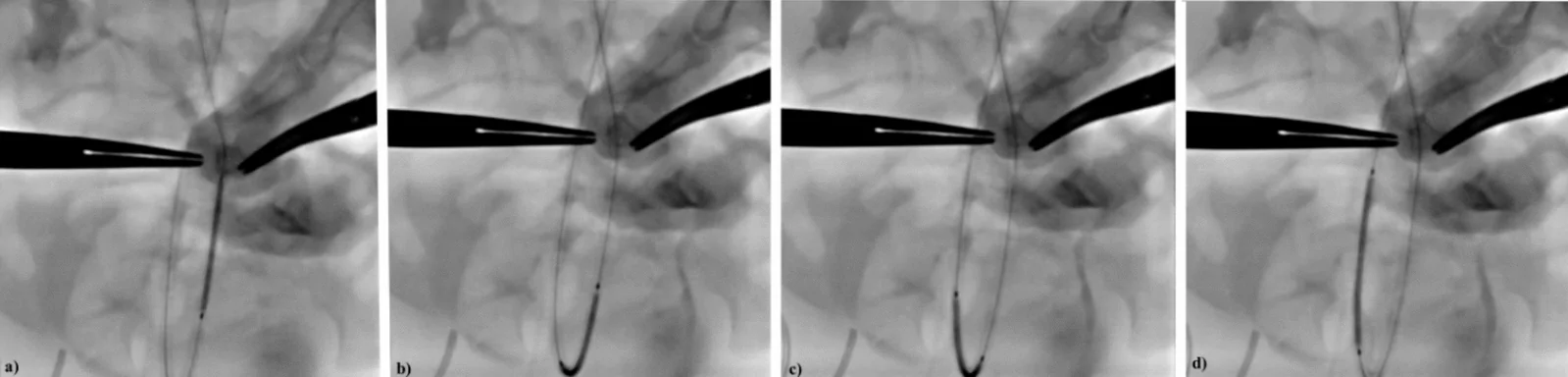
Demonstrates Umbilical access for PDA stenting; **a**–**d** Sequential fluoroscopic images illustrating the advancement of the stent through a tortuous downward and upward trajectory through the umbilical artery into the iliac artery. Note that two forceps were used on either side of the umbilical stump to pull it up, facilitating the stent's progression through the long sheath along the tortuous course

During umbilical access, it was essential to lift the umbilical stump to allow the catheter to follow the umbilical artery's natural downward and upward curve as it transitioned into the iliac artery. Maintaining gentle upward traction on the stump while advancing the sheath minimized resistance and ensured smoother manipulation. (Fig. 2, video 1).

### Twisting/Locking Technique

**Fig. 3:**
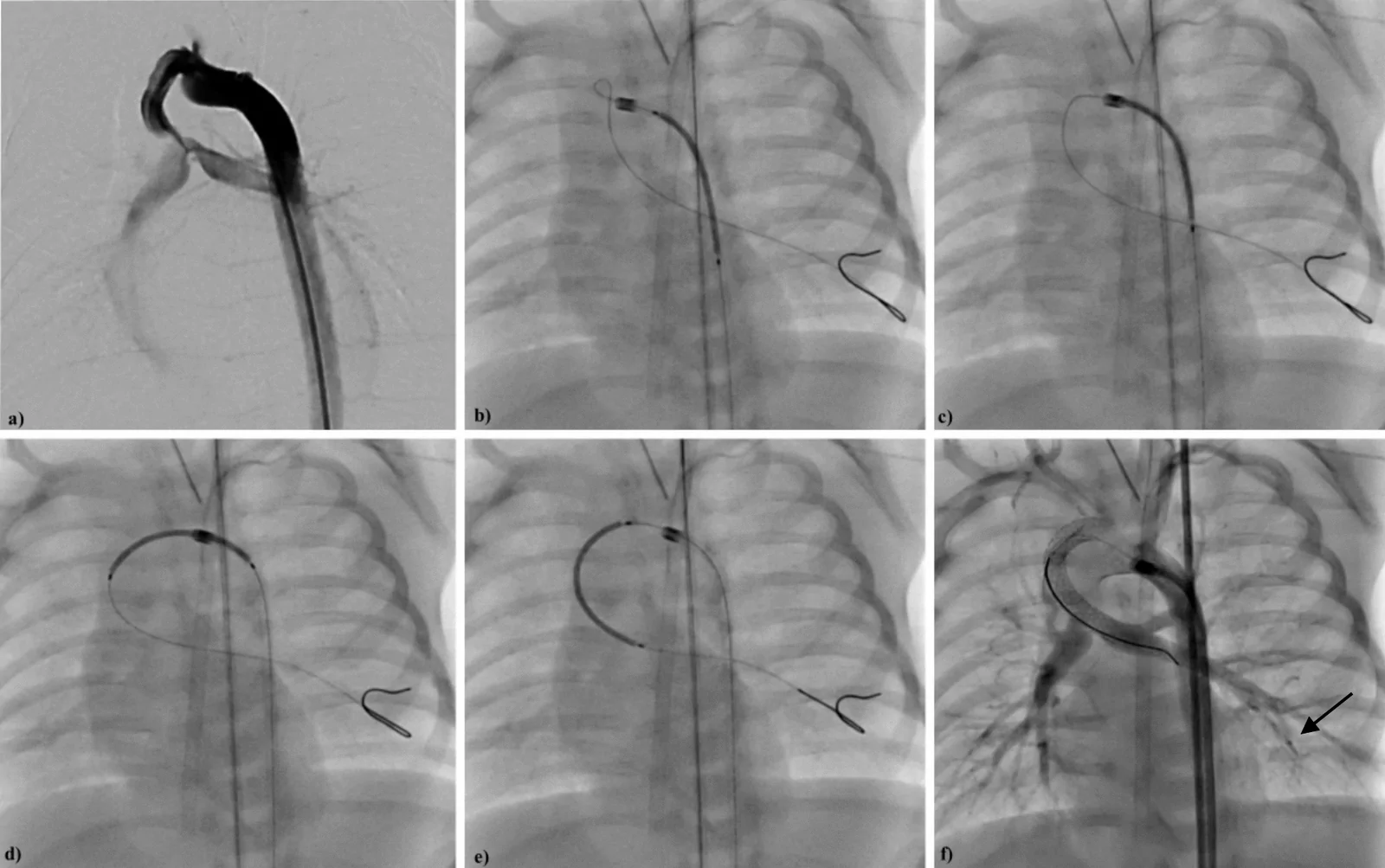
**a** Aortogram in the anteroposterior projection showing a PDA originating from the innominate artery, with stenosis at the pulmonary end of the PDA and the proximal right and left pulmonary arteries. **b**, **c** A 4 F-long sheath is positioned in the aorta at the aortic end of the PDA, with a BMW wire anchored in the distal LPA. Note the soft wire becomes straighter when pulled, increasing stiffness. **d**, **e** A long PDA stent is advanced over the BMW wire, with varying wire tension to navigate the curve effectively. **f** Post-deployment angiogram in the aorta shows the PDA stent securely positioned with minimal repositioning as the wire is withdrawn. The microcatheter remains in place (the arrow indicates the microcatheter tip)

Once the Renegade and BMW wire were as distal as possible, we secured the wire by twisting it clockwise six times to lock it securely in place. We then tested the wire’s stability by tugging on it gently; if the Renegade advanced over the wire, we confirmed the lock was secure. The Renegade was then removed, leaving the wire in place. (Fig. 3).

### Stent Deployment

**Fig. 4:**
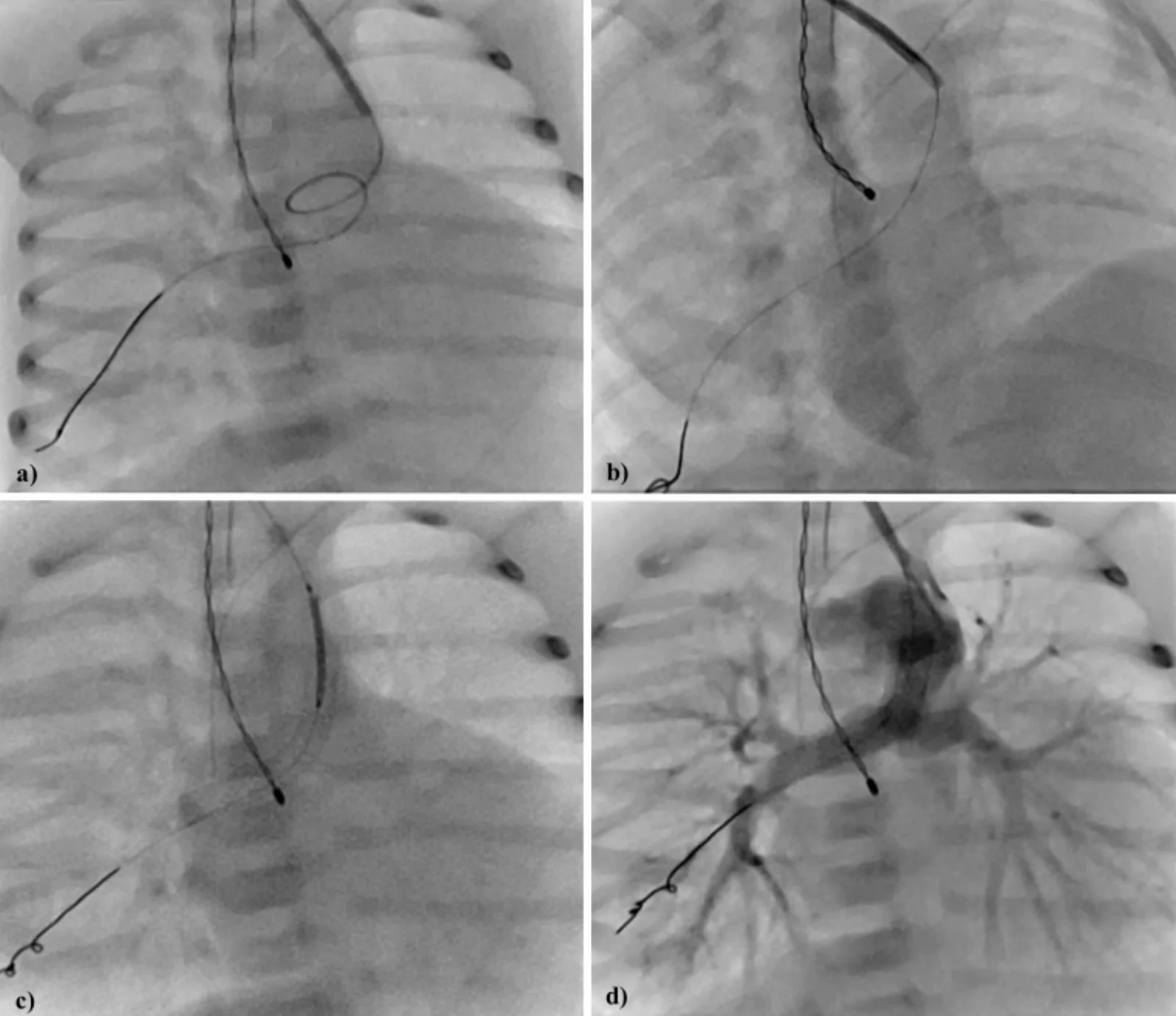
**a** A 4 F short sheath is placed in the right carotid artery. A microcatheter and BMW wire are advanced across the PDA, demonstrating the wire's ability to conform to the ductal anatomy without causing tension or spasms. **b** The wire twisting/locking technique is employed to increase the wire’s stiffness, enabling controlled navigation and precise stent positioning. **c** A second stent is deployed through the first stent to ensure complete coverage of the aortic end. Adjusting the wire tension (pulling it while its distal end is locked) allows the centering of the second stent through the first stent. **d** Final angiographic confirmation shows appropriate stent placement with adequate aortic and pulmonary ends coverage

An Onyx Frontier stent previously known as Resolute Onyx (Medtronic, Minneapolis, MN, USA) was inserted over the wire and advanced into position within the PDA. During stent placement, we adjusted the wire’s stiffness by altering its tension: releasing the tension softened the wire while increasing the tension made it stiffer (Fig. 4). This tension adjustment allowed for easier stent advancement/tracking and precise stent positioning across the PDA. Once positioned, we relaxed the wire, allowing the stent to conform to the PDA's natural shape. We then inflated the balloon to expand the stent. Subsequently, we performed an angiogram through the short sheath (or the long sheath in case of umbilical or femoral access) to confirm that the stent effectively covered both the aortic and pulmonary ends. If one end was not covered, we placed a second stent over the wire, utilizing tension adjustment to advance the second stent through the first one.

### Wire Removal

After confirming adequate stent placement, we reintroduced the Renegade over the wire into the distal vessel, ensuring the wire was not removed prematurely to prevent wire unraveling or injury to the lung parenchyma. Then, the wire was rotated counterclockwise six times, unlocking it from its torqued position. The wire was removed from the Renegade microcatheter. Then, a repeat angiogram was performed while the Renegade was still in place to ensure the stent position was unchanged after the wire was removed. Finally, the Renegade microcatheter was removed, and an angiogram was performed through the sheath.

We air on placing a longer stent which frequently jails one of the pulmonary artery branches, despite that we do not perform strut dilation during the initial stent implantation due to concerns about manipulating a freshly placed stent, which could increase the risk of stent displacement or vessel injury.

### Post-op Follow-up

All patients were monitored in the CTICU and remained intubated. At the end of the procedure, a loading dose of Plavix (2 mg/kg) was administered via nasogastric (NG) tube, followed by daily Plavix (1 mg/kg) and ¼ tablet of baby aspirin starting the next day. The duration of intubation varied based on the access site; patients with carotid or axillary access remained intubated for at least 4 h to minimize the risk of bleeding with movement.

### Statistical Analysis

Categorical variables were reported as frequency and percentage and continuous variables were represented as median with interquartile range (first quartile–third quartile).

## Result

The wire twisting/locking technique was applied in 18 PDA stenting procedures performed on 11 patients with ductal-dependent pulmonary circulation including double-inlet left ventricle (DILV) (*n* = 3), double-outlet right ventricle (DORV) (*n* = 3), pulmonary atresia with intact ventricular septum (PA/IVS) (*n* = 3), and complete AV canal with pulmonary atresia/stenosis (*n* = 2). The median gestational age was 37 weeks (IQR: 34–39 weeks), and the median birth weight was 3.03 kg (2.65, 3.35). Two unstable patients with PAIVS required extracorporeal membrane oxygenation (ECMO) before the procedure. Baseline patient data, including syndromic conditions, pre-procedural SpO_2_, PDA size, and surgeries, are summarized in Table 1.

**Table 1:** Patients characteristics

Patients’ No	Sex	Gestational age	Diagnosis	Associated lesions and comorbidities	Syndromic		Birth weight (Kg)		PDA length (mm)		PDA diameter (mm)		Surgery		Initial Sat (%)
1	F	39w + 1	DORV, PA	Bronchomalacia requires bronchial stent		–	3.03	18.3		2.4		Awaiting complete repair		90	
2	F	38w + 2	PA/IVS	–		–	3.41	17.8		1.9		–		85	
3	M	34w	PA/IVS	ASD		Microarray	1.8	23.4		2		Heart transplantation		70	
4	M	37w + 5	DORV, PS	Dextrocardia, malposed great arteries, high output heart failure		–	3.35	15.7		1.3		Awaiting complete repair		59	
5	M	39w + 2	DILV, PA	Dextrocardia, stenotic left side AV valve		–	3.56	30		2.6		Glenn		85	
6	M	30w + 4	Unbalanced AVC, PA	D-malposed great arteries, ASD, VSD		Vacteral	1.12	12.2		2.2		Awaiting complete repair		74	
7	F	34w + 3	CAVC, PA,	TAPVR		Heterotaxy	2.85	20.7		1.5		TAPVR repair & Glenn		60	
8	F	37w + 2	DILV	ASD/VSD		–	1.89	18.5		1.4		–		83	
9	M	38w + 3	DILV, PA	Dextrocardia		–	3.84	16.4		2.1		Glenn		80	
10	F	37w + 2	PA/IVS	ASD		–	3.27	16.2		2.6		–		72	
11	F	38w + 6	DORV, PA	L-looped ventricles, right-sided mitral valve hypoplasia, LV hypoplasia			2.95	12.4		1.98		Awaiting complete repair		67	

*DORV* double outlet right ventricle, *PA* pulmonary atresia, *VSD* ventricular septal defect, *ASD* atrial septal defect, *IVS* interrupted aortic arch, *BSA* body surface area, *DILV* double inlet left ventricle, *PS* pulmonary stenosis, *TAPVR* total anomalous pulmonary venous return, *AVC* atrioventricular canal, *DROV* double outlet right ventricle, *LV* left ventricle, *RV* right ventricle

The procedures included initial PDA stenting (*n* = 10) and PDA stent re-interventions (*n* = 8), comprising four stent placements and four balloon dilations. In one patient, the twisting/locking technique was not used for their initial PDA stent placement but was employed in a subsequent re-intervention of the existing stent. The reason for re-intervention was intimal buildup in two cases. In the remaining patients, the stent was dilated to accommodate for growth and to delay surgery till patient was older. Importantly, none of the re-interventions were performed for PDA spasm or stent thrombosis.

Eight PDAs (73%) originated from the undersurface of the transverse aortic arch, while 3 PDAs (27%) arose from the innominate artery. The PDA type distribution included Type 3 in 5 patients (45%), Type 2 in 5 patients (45%), and Type 1 in 1 patient (9%).

Vascular access sites varied based on the patient’s anatomy and PDA orientation, with the use of carotid (*n* = 10), femoral (*n* = 4), axillary (*n* = 2), and umbilical arteries (*n* = 2). The same vascular access used for initial stenting was used for re-intervention, except in the two that had umbilical artery access for the initial stenting, those two had femoral artery access for the re-intervention procedure.

The median procedure time was 91 min (IQR: 62–130 min). The median radiation dose was 154.73 Gycm^2^ (IQR: 96.21–349.83 Gycm^2^), while the median fluoroscopy time was 26 min (IQR: 14–40 min). The median stent diameter was 3.5 mm (IQR: 3.5–4.5 mm), and the median stent length was 24.35 mm (IQR: 22–30 mm). Initial stent placement consisted of a single stent in 8 patients and two stents in 2 patients. (Table 2).

**Table 2:** Procedure details

Age at first initial catheterization Weight at first initial catheterization		9 days (IQR: 8–90) 3.88 kg (IQR: 3.17–6.05)
PDA origin (*N* = 11)		
	*Under transverse aortic arch*	8
	*Innominate*	3
PDA type (*N* = 11)		
	*Type 3*	5
	*Type 2*	5
	*Type 1*	1
Reason for catheterization (*N* = 18)		
	Initial PDA stenting	10*
	• *Single stent*	8/10
	• *Double stent*	2/10
Reintervention		8
	• *Ballon dilation*	4/8
	• *Re-stent*	4/8
Access (*N* = 18)		
	*Carotid*	10
	*Femoral*	4
	*Axillary*	2
Wire locked-in (*N* = 18)		
	*RPA* *LPA*	13 5
Stent diameter (mm)		3.5 (3.5–4.5)
Stent length (mm)		24.35 (22–30)
Procedure time (Min)		91 (61–130)
Fluoroscopy time (Min)		26 (14–40)
Radiation dose (Gycm2)		154.73 (96.21–349.83)
Discharge sat O2(%)		85 (82–89)

^*^In one patient the twisting/locking technique was not used for their initial PDA stent placement but was employed in a subsequent re-intervention in the form of balloon dilation of the existing stent

^**^Access sites used for re-intervention were the same as those used for initial PDA stenting except those with umbilical access for the initial PDA stenting where femoral access was used for re-intervention

Post-procedure, patients with axillary or carotid access remained intubated for at least four hours to reduce bleeding risk from movement, with most extubated within 24 h. Two patients required prolonged intubation due to complex comorbidities: one with a PDA stent as a bridge to heart transplant and another, medically complex patient who required a tracheostomy.

No intra- or post-procedural complications were observed, particularly no lung injury or hemoptysis associated with the wire twisting/locking technique. There was no PDA dissection, thrombosis, or stent migration.

## Discussion

PDA stenting is increasingly recognized as the preferred approach for establishing pulmonary blood flow in cyanotic infants with ductal-dependent pulmonary blood flow [7, 9, 17–19]. Several studies have demonstrated excellent success rates, even in highly tortuous PDAs [3, 20]. However, devastating complications have also been reported, including stent malposition, migration, ductal dissection, and acute stent thrombosis [2, 3, 5]. Reintervention following PDA stenting is common due to various reasons, such as intimal buildup or stent fracture [14]. Planned re-interventions can also extend the lifespan of the PDA stent and delay the need for surgery, effectively treating the PDA stent as a “growing shunt” [21]. That said, re-interventions and stent re-crossing can be challenging, especially when the stent protrudes at the aortic end—a design often necessary to prevent constriction at the aortic end [22]. Delivering a stent through a previously placed stent can be particularly complex, as noted in coronary artery re-stenting [23], and similar challenges have been observed during PDA stent interventions. Reintervention is further complicated by tortuous stent paths, jailed or crossed branches, and stent fractures. These difficulties underscore the importance of proper guidewire placement in the distal pulmonary artery branch; inadequate placement has been identified as a major cause of procedural failure, leading to complications like acute stent thrombosis or dislodgment [24].

The wire twisting/locking technique was first described by Linnane et al. in the context of RVOT stenting [12]. Kato et al. subsequently adapted this technique for PDA stenting via the femoral approach, using a 5 F sheath and guide catheter in the femoral artery [13]. Our study employed this technique for PDA stenting via multiple access points, including carotid, axillary, and umbilical arteries, using a 4 F system instead of a 5 F one. This report includes 18 procedures in 11 patients using the wire twisting/locking technique. By securing the guidewire in the distal PA or lung parenchyma, smooth stent navigation to the target site was achieved without complications, even in cases requiring PDA stent re-intervention.

Careful technical planning is crucial for complex cases, particularly those involving a tortuous duct or challenging ductal origins. Some experts recommend multiple stents for total coverage [25], while others advocate for a single long stent to minimize risks [10]. Utilizing multiple short stents increases the likelihood of dislodging the previously placed distal stent [26, 27]. In our study, a single long stent was predominantly used, allowing us to mitigate the risk of migration and displacement. While shorter stents are easier to navigate through tight curves, the wire twisting/locking technique enabled us to navigate a longer single stent through complex curves without issue. Most patients in our study had a high tortuosity index (type 2 and 3), and in all but two cases, a single long stent was placed without complications. In the remaining two cases, an additional short stent was required to ensure adequate aortic end coverage. The wire twisting/locking technique facilitated seamless navigation of the second stent through the first, ensuring proper overlap without dislodging the distal stent.

None of the re-interventions in our series were performed for thrombosis. All re-interventions were related to patient growth or intimal buildup occurring several months after the initial procedure, not due to acute stent-related complications.

Prior studies have suggested the use of stiff wires, such as Choice PT (Boston Scientific, Marlborough, MA, USA) or Iron Man (Abbott, Santa Clara, CA, USA), to help straighten the PDA [28, 29]. However, these wires carry risks, including ductal spasm, dissection, and stent migration upon wire removal [4, 30]. The wire twisting/locking technique provides dynamic stiffness adjustment by altering wire tension: releasing tension softens the wire while increasing tension stiffens it. This adaptability allowed us to fine-tune the wire’s stiffness and predict the final PDA stent configuration.

A key distinction between our study and the work by Linnane et al. [12], who employed this technique for RVOT stenting, lies in the choice of wire. While the RVOT study utilized the rigid Choice PT wire, we opted for the more adaptable BMW wire, enabling real-time stiffness adjustments.

Acute stent thrombosis that has been reported in 2–3% of cases, is a critical complication with potentially catastrophic outcomes [6, 7, 30]. We believe that our technique may have mitigated this risk by avoiding the advancement of stiff wires that can result in abrupt straightening of the PDA, which can induce twisting or ringing of the PDA. Instead, we used the BMW wire and Renegade microcatheter, to safely cross the ductus. Furthermore, eliminating the need for wire exchange reduced the risk of wire loss or ductal spasm from re-crossing, which can lead to sudden desaturation requiring immediate management [6, 28]. We believe that using a soft wire with precise tension adjustment may have helped avert these complications in our patients.

Haddad et al. reported the use of a steerable microcatheters, SwiftNINJA (Sumitomo Bakelite, Tokyo, Japan), as a promising alternative for PDA engagement and deep anchoring. They described that its use allowed real-time tip articulation, facilitating precise navigation through tortuous PDAs [31].

Our findings highlight the importance of tailored strategies to optimize PDA stenting outcomes. By employing the wire twisting/locking technique and favoring a single long stent, we believe we effectively addressed anatomical challenges, minimized complications, and enhanced procedural safety. Further studies are warranted to validate these findings in larger cohorts and assess long-term clinical outcomes.

## Conclusion

The twisting/locking technique represents a promising modification for PDA stenting. Utilizing a dynamic soft wire and opting for a single long stent simplifies stent placement and minimizes procedural complications.

## Limitations

This study has several limitations. The small cohort and single-center design limit generalizability to broader populations or settings. We acknowledge that twisting the wire could theoretically result in lung injury, including bleeding, and this is something we are vigilant about during the procedure. However, we have not encountered this complication in any of our patients to date. The technique’s learning curve and reproducibility across less experienced operators remain untested. Finally, the retrospective design lacks a control group (e.g., standard stenting), preventing direct comparison of efficacy and safety. These constraints position this study as a proof-of-concept, requiring larger, multicenter trials for validation.

## Supplementary Information

Below is the link to the electronic supplementary material.

Supplementary file1 (DOCX 14 KB)

Supplementary file2 (MP4 10,762 KB)

Supplementary file3 (MP4 9108 KB)

## Data Availability

No datasets were generated or analysed during the current study.
