# Supplementary material for: The Wire Twisting/Locking Technique to Facilitate Precise PDA Stent Delivery in Neonates with Ductal-Dependent Pulmonary Blood Flow
Source: Pediatr Cardiol. 2025 May 5;47(6):2405–14. doi: 10.1007/s00246-025-03852-2 (PMC13498504; doi:10.1007/s00246-025-03852-2)
Supplement: Supplementary file 1 [file 246_2025_3852_MOESM1_ESM.docx]

**Video 1**: This video illustrates the treatment of a complex single-ventricle patient with pulmonary atresia, focusing on 3D reconstructions of the PDA entering the LPA and angiographic techniques for stent placement. It demonstrates axillary access, a twisting locking technique to secure the wire, and precise tension adjustments to navigate and deploy a long PDA stent without causing spasms. The video concludes with the successful positioning of the PDA stent.

**Video 2**: This video demonstrates the **wire-locking technique** for PDA stenting. It shows **clockwise twisting to lock the wire** and confirmation by gentle traction. **Wire tension adjustment** is used to deliver the first stent, and a second stent is placed to cover the uncovered pulmonary end. Tension adjustments help center the second stent within the first. The video concludes with the **final stent positioning** and **counterclockwise unlocking and removal of the wire**.
